# Objective consequentialism and the plurality of chances

**DOI:** 10.1007/s11229-020-02851-5

**Published:** 2020-09-02

**Authors:** Leszek Wroński

**Affiliations:** Institute of Philosophy, Faculty of Philosophy, Jagiellonian University, Grodzka 52, 31-044 Krakow, Poland

**Keywords:** Objective consequentialism, Reference class problem, Chance, Probability

## Abstract

I claim that objective consequentialism (OC) faces a problem stemming from the existence in some situations of a plurality of chances relevant to the outcomes of an agent’s acts. I suggest that this phenomenon bears structural resemblance to the well-known Reference Class problem. I outline a few ways in which one could attempt to deal with the issue, suggesting that it is the higher-level chance that should be employed by OC.

## Introduction

Objective consequentialism (OC), the approach to ethics according to which ’the primary notion for moral theory is given by what is best (or (...) has greatest objective value) regardless of how things seem to the agent’ (Oddie and Menzies 1992, p. 512) and ’the correct regulative ideal for the moral agent is that of maximising objective value’ (*ibid.*, p. 513), has been criticised in a number of familiar ways. Perhaps the best point of departure is Lenman (2000), where probably the most immediate objection to the OC, stemming from the obvious intuition about the difficulty of predicting the future, is discussed. In this paper I will present what I believe to be a hitherto overlooked problem the OC needs to face. It is somewhat similar in spirit to that posed by situations in which ’there is no fact of the matter about whether things will turn out well or badly if we do one thing or another’ (Hare 2011, p. 199); however, in the cases described below it seems there are too many relevant facts, pulling our ethical reasoning in conflicting directions.

The main idea I want to propose is that the objective variant of consequentialism faces an issue which is somewhat structurally similar to the well-known reference class problem, and that to this issue no clearly best solution presents itself. In a nutshell, one reading of the classical reference class problem takes it to be generated by the fact that an individual can be classified as belonging to various sets, and so where one is looking for a single probability value one finds a multitude of them instead. Modern philosophy of science takes seriously the idea of *chance plurality*, that is, that some propositions have, at a fixed moment in a fixed world, more than one chance value. In situations displaying chance plurality, one and the same proposition may have various chances—fundamental and higher-level ones. This might lead to objective consequentialism imposing contradictory obligations on some agents in some situations. While in my opinion accepting this should be seriously considered, I will suggest that the right version of objective consequentialism should lead to obligations based on higher-level chances. However, the argument for this requires some assumptions unacceptable to a pure conseqentialist. In effect, I argue that the right version of objective consequentialism needs to be ’impure’ in the sense that it should require one to care about more than just maximizing a value function. In my opinion pure objective consequentialism has no way out of the problems raised by chance plurality.

In what follows I will only be talking about act consequentialism. It is quite possible that similar issues also arise for rule consequentialism, but the matter is not entirely clear to me yet and I plan to explore this in a later paper.

## The gist of objective consequentialism

The main idea behind act consequentialism is that an act’s moral value stems only from its consequences as compared to the consequences of the other acts available to the agent; some way of evaluating the consequences needs of course to be assumed. There are at least two lines dividing the field of consequentialist approaches. One demarcates those according to which what matters are the act’s real consequences from those which take into account consequences considered from at least partially subjective point of view—for example, consequences which can be rationally predicted. This is the division between *objective* and *subjective* families of consequentialism. Another overarching distinction is that between those approaches which employ probability when comparing the values of acts—the *probabilistic* approaches—and those which do not. The nonprobabilistic approaches are usually *subjunctive*, if they resort to would-subjunctive conditionals^1^, or *possibilist*, if they use modal accessibility relations coupled with some possible-world-ranking system.^2^ As already stated, in this paper I am concerned with objective consequentialism. That its correct form is probabilistic, regardless of whether the world itself is deterministic or not, has been in my opinion conclusively established by Vessel (2007). I will be, though, describing a problem such an approach must face; should the Reader come to the conclusion that the issue is unanswerable properly, they might believe that it speaks for adopting a non-probabilistic form of consequentialism, or for switching to subjective consequentialism, or, finally, for moving away from consequentialism altogether.

Any probabilistic consequentialism claims that agents have an obligation to maximise expected moral utility, that is, to maximise the value of what we will call the ’consequentialist formula’ (CF for short):

$$\begin{aligned} \sum _{i}Pr(O_i | A _j) \times V(O_i), \end{aligned}$$

where the $O_i$’s are the possible outcomes, *V* is the consequentialist value function, *Pr*(*X*|*Y*) is the probability of *X* given *Y* (see below), the $A_j$’s are the agent’s possible acts, and, to be sure, maximisation is over the acts $A_j$.^3^ The act which maximises the formula is what the agent ought to do in the given situation.

I would like to stress that I am not suggesting objective consequentialism to be a sort of decision procedure; we should take it to be a criterion of rightness (see the beginning of Feldman 2006, and also Stark 1997, as well as references therein). We have an obligation to do the right thing. Consequentialism says that the right thing to do is the one which maximises the CF. According to the objective variant of consequentialism—which, as I have already mentioned, I take to be probabilistic in nature—objective chances, which might not be known to the agent, feature essentially in the CF. Therefore objective consequentialism cannot function as a decision procedure; it cannot in general guide agents in their choices. Rather, it specifies the right act, and by that the agent’s obligation, that is, what the agent ought to do, given the full set of data about the value function and relevant probabilities. It is therefore fully compatible with the idea that for some agents on some—if not many, or even most—occasions it is impossible to know what the right act is. However, this lack of knowledge does not, of course, annul the obligation to perform that act.

Just like in probability theory and statistics it is normal to say that a random variable has an expected value even if no subjectivity is involved anywhere, and no person really *expects* anything relevant to the topic at hand, the fact that consequentialism involves maximising expected moral utility does not make it subjective from the start. The division between the objective and subjective branches of consequentialism also does not hang on the notion of value: it is assumed that some preference ranking of the possible outcomes is in the background which allows a sensible numerical representation of the values. Whether consequentialism is subjective or objective depends, however, on the nature of probabilities involved. If $Pr(O_i|A_j)$ refers to an agent’s opinion as to how likely it is that the given act leads to the given outcome, or if this probability, while not referring to the agent’s opinion about likelihoods, still depends on the evidence available to the agent—that is, if the probabilities featuring in the CF are *epistemic* in nature—the consequentialism under discussion is *subjective*. If, on the other hand, those probabilities are *chances*, if they do not depend on what anyone thinks, but are generated by features of the physical world itself, the discussed consequentialism is *objective*. In the following sections I will describe a few problems plaguing probabilistic consequentialism, and most pressingly its objective variant.

The details of the CF and its possible variants will not concern us: in this paper I will not go through any calculations and my claims are not related to any peculiarities of mathematical formalism. The important, if trivial, thing to observe at this point is that in a specific situation, given a sphere of possible acts and values of the possible outcomes, probability is all that matters: what an agent ought to do depends completely on the probabilities. If the probabilities are not well defined, then consequentialism encounters a problem.

## The reference class problem

The reference class problem raises its head whenever we would like to practically apply probability. It seems that historically the most persuasive examples of the issue involved predictions concerning personal well-being. Suppose you’re a higher-educated American Caucasian female aged 35 living in Boston. You are ill and in need of a cure. There are two drugs for your illness, A and B, of which you can only take one. You would like to decide which of the two drugs to take. If you wish to base your decision on the fact which drug offers you the higher chance of recovery, what kind of statistical information should you defer to? If ’better among X’ means just ’offers a higher chance of recovery for members of X’, it might very well happen that drug A is better than drug B among females, and B is better than A among Caucasians. You may aim—following e.g. Reichenbach—to refer to the data concerning the narrowest reference class containing enough people for the relevant frequencies to be considered as probabilities. However, it is very much possible that among the classes for which data is available, there are two maximally narrow ones, in which the probabilities significantly differ: for example, drug A may be better than drug B among higher-educated female Caucasians living in Boston, while drug B may be better than drug A among higher-educated female Caucasians before 40. Assume that data regarding narrower classes is not available.^4^ Which class, then, is the one which determines the probability of interest? Even calling upon the best available data regarding causal relevance might not be enough to fix a single class.

It is clear why the issue is called the *reference class* problem: an individual can be correctly classified as belonging to a number of groups which determine different probabilities of their possessing a certain trait; if we are interested in *the* probability that the individual possesses such a trait, we need to fix a unique reference class. The issue is frequently discussed in the legal literature, in the context of procedures which demand an estimation of some quantities on the basis of statistical data, for example the most probable quantity of drugs which was smuggled by a drug mule during a specified time period, when the mule can be classified as a drug smuggler caught at the JFK airport, a Nigerian drug smuggler, and in a number of other nonequivalent ways (Cheng 2009). General practical solutions to the issue may require employing concepts outside of the realm of probability and statistics, and talking for example about mechanisms—for a recent survey of possible approaches see Wallmann and Williamson (2017).

Hájek (2007) argues that the problem arises regardless of the probability interpretation we might adopt: it would seem there should be one, unconditional probability of *X*, yet what we can find are different conditional probabilities of *X* given various propositions. *X* has differing probabilities depending on how it is relativized, but none of the conditions to which the relativization is made presents itself as *the right one*. I do not want to engage here in the subtleties of the problem and its proposed solutions, but treat it as an illustration of a more general phenomenon regarding what I’d like to call ’probabilistic definite descriptions’: expressions of the form ’the probability that *X*’. There is nothing abnormal about such expressions; in fact, it is hard to talk about probability without using them. However, the reference class problem shows that this straightforward talk masks the need for a parameter the value of which has to be fixed before the expression successfully refers. Insofar as the OC requires the use of expressions of the sort ‘the probability that act *A* leads to the outcome *O*’, it may be beneficial for its proponents to acknowledge the existence of the problem. However, I do not want to suggest that it is an issue it would be their job to solve, since, as already mentioned, the problem has been extensively studied. What I do want to point out is that there is a structurally similar problem which the OC needs to take seriously, which stems from the phenomenon of chance plurality, to which we now turn.

## The plurality of objective chances

One of the topics in philosophy of science which seems to have gained prominence since the later aughts is that of objective chance and, in particular, whether it makes sense to speak of non-trivial objective chances in deterministic worlds. One of the most cited papers on the topic is (Schaffer 2007). The author argues that determinism and objective chance are incompatible, first identifying a set of key principles making up the ’role of chance’, that is, the connections between chance and credence, possibility, causation, and a few further notions. For example, chance should direct rational credence: if no additional interfering evidence is received, information that the objective chance of *p* equals *x* should fix a rational agent’s credence in *p* to *x*. One important relation between chance and possibility is the fact that non-zero chances denote possibility: if there is a higher than zero chance that *p*, then it is possible that *p*. The connection with causality concerns chances which are used to explain some transition from a cause to an effect and restricts the range of temporal affixments of such chances: roughly, they have to be chances at moments between the time of the cause and the time of effect. Schaffer formulates in total six substantive principles and proposes that chance is what ’best satisfies’ those principles, as well as some relatively noncontroversial formal conditions (Schaffer, *ibid.*, p. 126). He then offers detailed arguments that in a fundamentally deterministic world the chance functions can obtain only the values 0 and 1; in a deterministic world there are no non-trivial objective chances.

A number of authors, including for example Glynn (2010), Frigg and Hoefer (2015) and Emery (2015) observed—contrary to Schaffer—that in deterministic setups a non-trivial probability function can play the role of chance; it seems that it can very well satisfy all the principles identified by Schaffer, and there would be little point in insisting that it should not be called ’objective chance’. (Making such a point requires, obviously, switching from talking about chance as the unique thing which ’best satisfies’ the above-mentioned set of principles to talking about chance as one of possibly many things which satisfy those principles maximally well.) The usual examples depict situations in which a deterministic low-level, or ’micro’, chance function is accompanied by a non-trivial objective higher-level, or ’macro’, chance function. The point is, however, generalised for example in (List and Pivato 2015) both fundamentally deterministic and fundamentally indeterministic worlds can feature both trivial and non-trivial objective chances at higher levels.

The Reader may be familiar with a popular outlook (probably inspired by Laplace) combining a fundamental determinism with the belief that any non-trivial probability assignments stem from our lack of knowledge about the inner workings of the world. Even disregarding doubts regarding this germinated by post-Newtonian physics, one could think that due to our cognitive imperfections it is impossible for us to know the whole state of the universe, but it is that whole state which uniquely determines the universe’s temporal evolution. Any non-trivial probability assignments are then, supposedly, epistemic in nature, corresponding to this lack of our knowledge. It needs to be stressed that a sizable part of the modern literature on objective chance in philosophy of science from which I have cited a few examples above consciously—and, in my opinion, correctly—negates this outlook: there are objective probabilities, in-the-world chances, at all levels under discussion.

Another of the principles Schaffer took to be constitutive of the role of chance took it to be codified by natural laws. Most of the examples familiar from the literature of objective chance which feature different chances at different levels of description either originally explicitly feature laws or can be, I think, naturally rephrased so that laws are mentioned. As illustrations of multiple level-relative chances consider the following examples (inspired by Glynn 2010; List and Pivato 2015, who discuss similar cases, sometimes with more, sometimes with less detail):^5^

- *Diffusion*: a rectangular container is filled with water. A portion of a dye is introduced to the container in one of its corners. We are interested in the chance that after 10 s more than 1 ml of the dye will be present in the liquid in the part of the container defined as being closer than 1 cm to the corner opposite to the one at which the dye was introduced. The deterministic laws of classical mechanics will give us one value of that chance, and the statistical laws regarding diffusion will give us another.
- *Dice*: a pair of dice is to be thrown on a gambling table. Suppose that some ’randomising’ apparatus is to be used—for example, the gambler is supposed to push a button turning on on a machine which will rotate a special tube containing the dice (constructed, say, according to the schematics from the patent application US20110193288A1).^6^ What is the chance that on a particular occasion the gambler is about to throw snake-eyes in this way? Again, the complete microphysical description of the state of the tube and the throwing machine coupled with the deterministic laws of classical mechanics dictate one answer: 1 or 0. However, that same gambling system, being a device for random throws of two fair dice, admits a higher-level description according to which the chance is 1 in 36.
- *Genetics*: consider two binary plant traits. Suppose two plants are to be crossed which are heterozygous for each of the two traits. Consider a plant which is the product of this crossing. What is the chance that this particular plant will be homozygous for both traits? The laws of Mendelian genetics dictate the answer of 0.25; if the fundamental laws of our world are deterministic, they either dictate the chance being equal to 1 or dictate it being equal to 0.
- *Meteorology*: suppose the atmosphere of our planet admits a description at the microphysical level which is fully deterministic. However, the description of future weather events meteorologists—and we—are interested in belongs to a higher-level language, in which predictions are formulated which typically follow from statistical laws. A proposition concerning such an event, say, that at least one drop of rain falls tomorrow in Munich, is assigned an objective chance of 0 or 1 by the microphysical laws and a non-trivial objective chance by the statistical meteorological laws.

A somewhat natural reaction to some of these examples may be to say that it would be beneficial to apply the crucial distinction between a concrete physical system in a particular microstate *s* and a type of systems classified as being in a macrostate *S* which can be realised by a multitude of microstates, including *s*. One could attempt to claim that the two chance functions corresponding to the two levels of descriptions have distinct domains: propositions about individual systems and propositions about system types. I would like to stress here that this would be a mistaken route: in the examples given we are talking about two different chances *for the same proposition*. Consider a proposition p regarding a future possible observable phenomenon, a trait a particular system can possess, described in a macro-level language. Then, it is perfectly possible that, for distinct values of *x* and *y*, *that same physical system* is at the same moment both (1) in a microstate governed by low-level laws which dictate a chance *x* of temporal evolution of that system which makes *p* true, and (2) in a macro state governed by higher-level laws which dictate a chance *y* of temporal evolution of that system which makes *p* true. That is, there are two distinct chances that *p*. If this is true for a system and some proposition *p*, I propose we say that the system exhibits *chance plurality*. We can sum this up in an informal definition, availing ourselves of the notions of 1) a physical system, 2) a proposition which is about a given physical system, and 3) level-relative chances:

- *Chance plurality*. A physical system exhibits chance plurality if and only if there is a proposition *p* about it and distinct level-relative chance functions $ch_l$ and $ch_L$ such that $ch_l(p) \ne ch_L(p)$.

In such cases it is meaningless to speak of *the* objective chance that *p*. Just like in the case of the reference class problem discussed above, fixing of an additional parameter is needed before ’the probability that *p*’ is defined. In the original problem the parameter was, perhaps obviously, the reference class. Here the parameter is the level of description (of course, specifying it will not tell us which reference class to use on that level: the previous problem may still raise its head!). In science the issue is not likely to be of pragmatic relevance since the scientists working in the particular field will be interested in the higher-level laws, the low-level laws not being practically applicable: the geneticist will use the statistical laws of Mendelian genetics, the synoptic analyst will use the statistical laws of meteorology, and so on. Still, the issue is there: in situations of chance plurality, the question ’what is the objective chance that *p*?’ does not have an answer.

## Objective consequentialism and chance plurality

Chance plurality may not be an aggravating problem for scientists, however, it raises an issue for subscribers to OC. Since the crucial formula CF employs objective chances, it might happen that it is not well-defined: depending on which level of description is used, different acts may be right, that is, different oughts might be ascribed to the agent.

To continue the above example involving dice, suppose that John, a gambler, formerly on a losing streak, has honestly decided in the morning to give all his winnings from that day to charity. For ease of exposition assume that he plays exclusively at a certain casino and only takes part in a single game of dice, in which snake-eyes doubles the player’s profit, any other outcome means that the casino takes all, the player can quit after any throw, and the casino can decide before any throw other than the first that it will be the last one. To his amazement the machine referred to above—working correctly, as certified by legitimate experts—has thrown snake-eyes a few times in a row, amassing a sizable sum of money for charity. The machine is set, waiting only for a push of the button. The casino decides the next throw will be the last one, if John decides to take it. Having integrated, in the true consequentialist spirit, any deliberations regarding risk, the non-linear value of money and so on into the value function, assume there are three outcomes:

- $O_1$, where the charity receives the money John has earned so far; assume $V(O_1)=1$;
- $O_2$, where the charity receives twice the money John has earned so far; assume $V(O_2)=1.5$;^7^
- $O_3$, where the charity receives nothing;

two acts:

- $A_1$, where John takes the throw (pushes the button);
- $A_2$, where John declines and the game ends;

and two chance functions:

- $ch_m$, the micro-level, deterministic chance,^8^ according to which the chance of one outcome of the throw is 1 and the chance of any other outcome is 0;
- $ch_M$, the macro-level chance, according to which the chance of any outcome of a throw of a fair die is 1 in 6.

Assume that the distribution of microphysical properties in the gambling system is such that, in fact, if the button is pressed, snake-eyes will be thrown: $ch_m(O_2| A_1)=1$. The act $A_2$ implies the occurrence of the outcome $O_1$; therefore, since chances are probability functions, regardless of which level of description is used, the expected moral utility of act $A_2$ is 1. However, regarding the act $A_1$ the situation is slightly nontrivial. This is because:

- $$ch_m(O_1| A_1)=ch_m(O_3| A_1)=0$$, while $$ch_m(O_2| A_1)=1$$;
- $$ch_M(O_1| A_1)=0$$, $$ch_M(O_2| A_1)=1/36$$, and $$ch_M(O_3| A_1)=35/36$$.

It is a matter of trivial calculation that the CF assigns the act $A_1$ higher expected moral utility than the act $A_2$ if $ch_m$ is used in the calculation and the act $A_2$ is assigned higher utility than $A_1$ if $ch_M$ is used. In other words, one thing is the right thing to do for the agent if OC uses the micro-level chance, and another thing is the right thing to do if the macro-level chance is used. In still other words, the agent ought to do one thing under OC using the micro-level chance, and ought to do another under OC using the macro-level chance: before some choice is made regarding this matter, OC prescribes the agent conflicting obligations (or no obligation at all, if we take the CF not to be defined).

The importance of such problems for the prospects of OC depends on how often chance plurality is displayed in situations involving ethical evaluation. If you believe that it is a phenomenon only occurring in rigorously controlled and precisely described physical environments, you will probably not be moved by the fact that on such occasions it makes OC contradictory or silent. However, if you take chance plurality, at least chance *duality*, to be a common occurrence, for example because in most if not all examples you can think of the distinction between the macro- and microlevels is intuitively clear, then you should believe that the issue of chance plurality is of serious concern to anyone subscribing to OC—just like the reference class problem is of critical import for anyone employing probability in legal reasoning. Depending on whether chance plurality is common or not, or if it is indeed ubiquitous, some solutions we will discuss will also look more or less reasonable.

I suggest that most chancy situations are describable on at least two levels involving laws of chance. Therefore, I give high credence to the following statement: in many situations in which an agent chooses between acts at least one of which is compatible with more than one outcome, and which outcome is eventually realised depends at least partially on chance, chance plurality is exhibited. Whether this leads to frequent problems for OC depends on how often chances meaningfully differ between the various levels; however, I give high credence to the statement that if the fundamental laws are deterministic, and higher-level laws are not, problems for OC similar to the one just described indeed arise. What can we do about this?

### Digression: examples involving micro- and macro-level indeterminism

The Reader will have noticed that all examples of chance-plurality mentioned so far involved fundamental determinism; this is also the case with the example about John.^9^ This is not a coincidence—the still relatively sparse literature on level-relative chances contains few, if any, worked out real-life examples of nondegenerate chance on both the lower and the higher level. The prevailing opinion is that nothing stands *a priori* against such an option. Establishing that a situation displays two non-degenerate chances would require (at least) an argument that on both levels the six principles proposed by Schaffer are satisfied, which is bound to be a nontrivial task. I would not like for such examples to play the biggest role in my argument against the OC, since I would risk that even those otherwise sympathetic to the chance plurality idea would find them wanting. Still, not to give an impression that chance plurality as posing difficulties for the OC requires fundamental determinism,^10^ I propose the following sketches of two examples which make no similar assumption.

The first case starts with the following example from List and Pivato (2015, p. 141): suppose ‘the police in a big city wish to forecast crime rates in various neighborhoods, in order to organize effective patrols. (...) The police will have to treat patterns of crime as involving non-degenerate objective chance. The chance of various crimes happening will differ from neighborhood to neighborhood: there is a higher chance of petty theft and pickpocketing at the railway station than on a quiet residential street’. Note that this is supposed to hold ’whether or not there is some physical or neuropsychological level at which each individual crime is predetermined’. Choose, then, some appropriate neuropsychological level and assume that the individual crimes are *not* predetermined on it, but subscribe to some chancy laws. Suppose that, on some day, in view of the recent crime-rate increase in the railway station area the officer in charge of patrol assignment can choose to allocate more officers to that region—or to send additional officers to a hitherto peaceful residential block. Suppose the neighbourhood-level objective chance List and Pivato write about indeed assigns a higher chance of crime-related incidents to the railway station area. On the other hand, the particular configuration of the city’s residents’ locations and their conditions combined with their chancy laws of individual crime-related behavior leads, on that particular day, the lower-level chance of criminal incidents to be higher in the residential block in question. It is easy to see how this leads to a case similar to the one above, involving John, the gambler.

The second idea follows a suggestion of one Reviewer who in the context of the plurality of chances mentioned currency exchange rates. My example will involve covered interest arbitrage (Levinson 2006, p. 25). The individuals under discussion live in the US; the choice to be made is whether to buy American, French or British 1 year bonds. Assume that Steven has been successful in his ForEx trading endeavours; in fact, he has amassed a small fortune and has become quite smug about his financial predictive skills. This has infuriated his aunt Jemima, firm in her belief that such proceedings are a modern form of common thievery, to no end. It so happens that aunt Jemima dies, making Steven the sole executor of her will. Its terms—by means of which Jemima wishes to teach Steven a lesson—state that, regarding a sizable sum of money left by the aunt, on the noon of the day after the will is opened Steven is supposed to announce his decision whether to invest the whole sum in American, French or British 1-year bonds, while the investment is to be carried out after exactly 7 days.

The financial situation on the day Steven is supposed to make his announcement is as follows: the interest rates in the three countries together with the spot and future exchange rates dictate that investing in American bonds is clearly the least profitable option, while British bonds are slightly preferable profit-wise to the French ones. However, since people will be selling dollars for pounds and Euros, the spot dollar/pound and dollar/Euro rates will decrease, while the 1-year forward rate rate will increase. We can consider the micro-level of description of the financial market, involving individual buyers and sellers, and the macro-level, involving larger groups and bodies, on which the Market Forces operate. On the assumption that both of these levels involve chances, we can consider the situation in which:

- due to the Market Forces in operation (government decisions, bank policies etc.) the macro-chance of the British bonds ending up as the most profitable option in a week is higher than the chance that one of the two other options ends up as the most profitable;
- however, due to the particular configuration of individual buyers and sellers, it is the micro-chance of the French bonds ending up as the most profitable which is the highest among the three options.

It is hopefully easy to see how one can on that basis construct an example similar to the one from the last section.

As already mentioned, I would not like these examples to be the main illustration of my argument against the OC. My objective in this paper is not to push forward the research on chance plurality; establishing whether the two examples from this subsection really ‘work’ would require e.g. a careful examination of whether we can legitimately speak about probabilistic laws in the context of economics and neuropsychological basis of human behavior—tasks which are clearly outside the scope of the current paper. I suggest, therefore, that the Reader keep in his or her mind the example of John the gambler as the main illustration of my point.

## The options

To repeat, we are considering situations in which the CF employed by the objective consequentialism awards some act two different values depending on which objective chance function is used. The issue is especially pressing if the act maximises the CF according to one of the functions (that is, the agent ought to perform it) but does not according to the other (that is, the agent ought to perform a different, possibly incompatible, act). Assume there are only two relevant levels to be discussed. We seem to have three families of choices:

1. *No answer*. We could decide that the question whether an agent ought to perform that act or not has no answer at all.
2. ‘*Dideontism*’.^11^ Another option would be to, in a way, embrace the conflict and state outright that it is true that the agent ought to perform the given act and that it is false that the agent ought to perform the given act. In logic the position that some sentences are both true and false is called ’dialetheism’ (Priest et al. 2018). We may give this position in the context of objective consequentialism the name ’dideontism’.
  It needs to be noticed that in all situations in which the CF is usually invoked, if an act does not maximise it, another act does. And so dideontism will make the above claim about at least two acts: that is, for two acts $A_1$ and $A_2$, it is true that the agent should perform $A_1$, it is true that the agent should perform $A_2$, it is false that the agent should perform $A_1$, and it is false that the agent should perform $A_2$. We could add indeterminacy to this view and say that while it is true that the the agent should perform $A_1$, and it is true that the agent should perform $A_2$, it is not *determinately true* that the agent should perform $A_1$; likewise for $A_2$ (Williams 2017).
3. *One privileged level*. We could decide that from the levels of description to which the objective chance functions are assigned one is singled out as the one which determines the normative issues. Still, it is conceivable *a priori* that different levels could be normatively relevant in different situations: sometimes the microphysical one, and sometimes a higher one. Therefore, the options are:
  3.1. *(micro)* In any situation featuring chance plurality, OC should employ the lower-level chance.
  3.2. *(macro)* In any situation featuring chance plurality, OC should employ the higher-level chance.
  3.3. *(mixed)* Which of the chance levels should be used by the OC depends on the details of the given situation.

Option 1 seems wrong, since it is at least *a priori* natural to suppose that it will usually be possible to provide, for a given system, two levels of description giving rise to differing chance functions, making the situation in which an agent ought to act in a specific way too rare a phenomenon. Among many obvious disadvantages of option 2, apart from seemingly bringing us closer to the outlandish proposal from the current paper’s motto, one in particular comes to mind: already before indeterminacy is invoked, we seem to face an outright contradiction with ’Ought implies Can’. This is because for at least two conflicting acts *A* and *B* it is true that the agent should perform *A* and it is true that the agent should perform *B*, but it is impossible to perform both of these acts. If we claim, in response to this, that for any act it is not *determinately* true that the agent should perform it, and if we transfer this indeterminacy to the level of obligation, we can say that in the cases featuring chance plurality the agent does not have a determinate obligation to perform any of the given acts. This, however, does not alleviate the previous issue: while we avoid the contradiction with ’Determinately Ought implies Can’ (since there is nothing the agent determinately ought to do in such cases), the contradiction mentioned previously remains. This leaves us with option 3. As already mentioned, I believe it gives those who adhere to OC a way of dealing with the problem; still, perhaps a slight deviation from purity of its consequentialist aspect is required to hold that what I propose is indeed a solution.

Note first that neither option 3.1 nor 3.2 should be argued for using the Principal Principle (PP), even if we accept that by resorting to it we are, as just pointed out, abandoning the consequentialist purity of the position under discussion. We have already mentioned that according to one of Schaffer’s chance-related principles, chances guide rational credences. Writers are divided concerning various details concerning the PP (introduced in Lewis 1986 and widely considered as a promising candidate for a norm of rationality), but there is a relatively wide agreement that it is a consistency condition on rational agents, which requires one’s credences to be coordinated with one’s credences about chances.

For example, if *Cr* is the credence function of some rational agent (which we assume is a probability measure), if there are two possible chances for *X*: .4 and .3, and if that agent’s credences in that each of these is the actual chance of *X* are respectively $Cr(ch(X)=.4)=.3$ and $Cr(ch(X)=.3)=.7$, then the PP says that $Cr(X| ch(X)=.4)=.4$ and $Cr(X| ch(X)=.3)=.3$, which allows us to calculate that agent’s rational credence in *X*. Namely, by the law of total probability, $Cr(X)=Cr(X| ch(X)=.4) Cr(ch(X)=.4) + Cr(X| ch(X)=.3)Cr(ch(X)=.3)=.4 \times .3 + .3 \times .7=.33$. That is, the PP shows how a rational agent’s credence in *X* is coordinated with that agent’s credences in what the chances of *X* are. PP may do its work even if the information about chance is fundamentally unobtainable, and can never be learned by anyone: it requires that, say, $Cr(X| ch(X)=.4)=.4$, even if no one can ever learn whether $ch(X)=.4$ is true.^12^

It might seem that we could contemplate the move which would have us employ in the CF that chance function which is used in the PP. This, however, will not help the cause of the OC, because, due to chance plurality, there is no single function doing the job. Most formulations of the PP will inform us about conditional credences of rational agents: credences in a proposition *X* conditional on what the chance of *X* is. Many formulations also include other, ‘admissible’^13^ propositions among those given which the conditional credence in *X* is specified. Usually the formulation one ends up with uses formulas of the sort ‘$Cr(X| E \wedge ch(X)=.3 )=.3$’, where *E* is some admissible proposition. Now, for some arbitrarily chosen values of the micro-level chance function $ch_m$ and macro-level chance function $ch_M$, the PP will only tell us that (for admissible *E*’s) $Cr(X | E \wedge ch_m(X)=.3)=3$ and $Cr(X | E \wedge ch_M(X)=.4)=4$. It will be silent when it comes to the question of the rational value of $Cr(X |E \wedge ch_m(X)=.3 \wedge ch_M(X)=.4)$. We would need some nontrivial additional philosophizing to produce a version of the PP which could handle such cases. This would require tackling the hairy notion of admissibility and, in particular, whether propositions about one sort of chance are not inadmissible given propositions about the other sort of chance. This would take us beyond the scope of the current paper and I would like to conclude that, as it stands, the PP is of no help to the objective consequentialist.

I do not see an argument for 3.1 or 3.3 which would be worth mentioning. However, in my opinion there is a route to option 3.2 which is available to the objective consequentialist—however, it will require abandoning the consequentialist purity of the position, that is, it will require one to care about more than just maximizing a value function. The route involves the notion of blame. Let me say at the outset that it will probably be of little interest to those who subscribe to ’quality of the will’ accounts of blame (see e.g. Strawson 2013; Tännsjö 1995), or think that the phenomenon of ’blameless wrongdoing’ (Parfit 1986) is common. As a first step, observe that we typically blame an agent for an event if she or he was the cause of that event (Kenner 1967), or at least among the causes of that event. Then note that if causation is considered as difference-making (List and Menzies 2009), it is reasonable to think of it as a relation involving higher-level properties and entities (for a recent defense of this view see Fenton-Glynn 2017); it may then be plausible to claim that higher-level causation involves higher-level chances. Suppose an agent holding a gun faces a choice whether to shoot someone or not; the kind Reader is asked to fill the requisite details of the story in her or his favorite manner, assuming only that the possible victim ought not to be killed. Then if our agent decides to shoot, and kills, she or he will not be blamed for putting the system consisting of the gun, bullet and the victim in the precise microphysical state the temporal evolution of which leads to another state which is among the infinitely many micro-states realising the macro-state of the victim being killed by a bullet, but will rather be blamed for pulling the trigger.^14^ The sphere of (micro-)physical possibilities is infinite, while agential possibility (List 2014) is coarse-grained, at least for the simple reason that fixing the microphysical state of the relevant system is outside the agent’s control. If blame presupposes alternate possibilities open to the agent, then, since these are not found at the micro-level, it is the higher-level chances which should be of import to moral evaluation.

This does not however give us a bullet-proof solution to our problem; we should not say that for that simple reason it is the higher-level chances which are of ethical interest and should be employed by OC. I specifically set up the casino example above so that the choice the agent faces involves two options; one in which the microphysical state of anything is irrelevant (the agent takes the previous winnings), and the other, which hangs on the behavior of a system whose not only macro- but also micro-physical state is fixed—the agent’s choice is not compatible with many physical possibilities corresponding to various possible states of the dice-rolling tube, but requires the tube to be used in the state in which it had been set up.

It seems to me, then, that there are two general, hand-wavy approaches one can take. The first and preferable one would be to simply say that in general blame-talk is cause-talk, and since causation involves higher-level propositions, the chance level to be used in the CF should be the higher one.

The second avenue can be opened by pointing to the fact that knowledge about the micro-level chances relevant to the given situation is typically unobtainable, while in principle it should be possible to attain information about the macro-level ones.^15^ By not maximising utility according to the CF as employed by the OC the agent displays then either a lack of in-principle-achievable knowledge or a conscious act against that knowledge. However, the flaws of such behavior are related primarily to issues of epistemic rationality. That an agent is epistemically irrational does not necessarily mean that he or she is morally reprehensible. It is tough to justify the claim that in situations displaying chance plurality the agent has a moral obligation to possess the knowledge about the higher-level chances.

The first approach seems to me, then, to be the more promising of the two. At this moment it seems to me that no other solutions present themselves, and even those considered here require the proponent of OC to abandon the consequentialist purity of that position, by bringing blame into the equation. If one is unhappy with both proposals, then I suggest that in view of recent developments in philosophy of science objective consequentialism should be abandoned.

## Conclusions

In this paper I identified a problem for objective consequentialism generated by chance plurality—on some occasions there is more than one chance function which could be employed in the consequentialist formula used to calculate the expected moral utility. I have suggested that if we take into account how the notion of blame is used we can reach the conclusion that in such cases it is the the higher level chance that should be called upon—this, however, is not an option available to a pure consequentialist. The general validity of this approach remains to be seen.

## Data Availability

Not applicable.

## Footnotes

1 Subjunctive approaches have been originally employed by Moore (see for example p. 30 of Moore 2005) and by numerous thinkers since (for example Carlson 1995).

2 See for example Feldman (1975) and chapter 1.3 of Feldman (1986).

3 I am assuming the formula is perfectly understandable for anyone reading about consequentialism, but if one needs a reference, here is one: (Jackson 1991). Nothing of relevance for the current paper hangs on whether the maximum (given the specific formula, that is, after specifying the *Pr* and *V* functions, the relevant outcomes and acts) is unique; I propose we assume it always is, since we can then use the expression ’the right thing to do’. The Reader will be able to reformulate all that follows without the uniqueness assumption. I will also sometimes write about ’maxi- / minimising the CF’ instead of ’maxi- /minimising the value of the CF’.

4 I would like to thank one of the Reviewers for suggestions regarding this example.

5 I am not claiming that the authors would agree with my presentations of the examples. With that said, the example about Mendelian genetics is lifted almost straight from (Glynn 2010).

6 See https://patents.google.com/patent/US20110193288.

7 For the coming reasoning it will only be needed that $V(O_2)$ is not outlandishly—say, 36 times or more—higher than $V(O_1)$.

8 As one Reviewer pointed out, significant doubts can be raised whether the assumption of fundamental determinism of the gambling machine is compatible with John making an undetermined choice. My idea was that the setup described would suggest that we could speak, in the case under discussion, of a probabilistic nomological machine in the sense of Cartwright (1999). But more importantly, please note that the assumption of determinism is not essential for the example; as will hopefully be clear, whenever the micro-level chance differs from the macro-level chance, an example posing a serious problem for the OC can be described.

9 Although, as mentioned, the assumption can be loosened without losing track of the import of the example.

10 And following the suggestions of the two anonymous Reviewers.

11 I would like to thank one of the anonymous Reviewers for suggesting this term. The initial version of the paper used the term ’diareteism’.

12 See Bana (2016) for an interesting formal controversy about this point.

13 For an entry point to modern discussion on the issue of admissibility in the context of the PP, see Lewis (1994).

14 Although it might sound weird to say this, our agent might be blamed for putting the system in *a* microphysical state which belongs to the set of states the temporal evolution of which lead to states which belong to the set all elements of which realise the macro-state of the trigger being pulled. But the agent will not be blamed for putting the system in *the* microphysical state that actually obtained. I would like to thank one of the Reviewers for pressing me on that point.

15 I would like to reiterate here that we are talking about objective chances, not epistemic probabilities.
